# Identification of a distinct developmental and behavioral profile in children with Dup15q syndrome

**DOI:** 10.1186/s11689-016-9152-y

**Published:** 2016-05-06

**Authors:** Charlotte DiStefano, Amanda Gulsrud, Scott Huberty, Connie Kasari, Edwin Cook, Lawrence T. Reiter, Ronald Thibert, Shafali Spurling Jeste

**Affiliations:** Department of Psychiatry and Biobehavioral Sciences, Semel Institute for Neuroscience, University of California Los Angeles, Los Angeles, CA USA; Department of Human Development and Psychology, Department of Psychiatry and Biobehavioral Sciences, Semel Institute for Neuroscience, University of California, Los Angeles, CA USA; Department of Psychiatry, University of Illinois at Chicago, Chicago, IL USA; Department of Neurology, Department of Pediatrics, University of Tennessee Health Science Center, Memphis, TN USA; Department of Neurology, Department of Pediatrics, Massachusetts General Hospital, Boston, MA USA; Department of Neurology, Department of Psychiatry and Biobehavioral Sciences, Semel Institute for Neuroscience, University of California Los Angeles, Los Angeles, CA USA

**Keywords:** Duplication 15q syndrome, Autism spectrum disorder, Intellectual disability, Social communication, Adaptive functioning

## Abstract

**Background:**

One of the most common genetic variants associated with autism spectrum disorder (ASD) are duplications of chromosome 15q11.2-q13.1 (Dup15q syndrome). To identify distinctive developmental and behavioral features in Dup15q syndrome, we examined the social communication, adaptive, and cognitive skills in clinic-referred subjects and compared the characteristics of children with Dup15q syndrome to age/IQ-matched children with non-syndromic ASD. Behavior and development were also analyzed within the Dup15q group for differences related to copy number or epilepsy.

**Methods:**

Participants included 13 children with Dup15q syndrome and 13 children with non-syndromic ASD, matched on chronological and mental age, ages 22 months–12 years. In the Dup15q group, ten participants had isodicentric and three had interstitial duplications. Four children had active epilepsy (all isodicentric). Participants were assessed for verbal and non-verbal cognition, ASD characteristics based on the Autism Diagnostic Observation Schedule (ADOS), and adaptive function based on the Vineland Adaptive Behavior Scales (VABS). Group comparisons were performed between Dup15q and ASD participants, as well as within the Dup15q group based on duplication type and epilepsy status.

**Results:**

All children with Dup15q syndrome met the criteria for ASD; ASD severity scores were significantly lower than children in the non-syndromic ASD group. ADOS profiles demonstrated a relative strength in items related to social interest. Children with Dup15q syndrome also demonstrated significantly more impairment in motor and daily living skills. Within the Dup15q group, children with epilepsy demonstrated significantly lower cognitive and adaptive function than those without epilepsy.

**Conclusions:**

The relative strength observed in social interest and responsiveness in the context of impaired motor skills represents an important avenue for intervention, including aggressive treatment of epilepsy, early and consistent focus on motor skills, and intervention targeting joint attention and language within a play context, in order to build on social interest to further develop social communication abilities. Longitudinal research beginning in early development will elucidate the temporal relationships between developmental domains and neurological comorbidities in these children at high risk for neurodevelopmental disorders.

## Background

Precipitated by rapid advances in molecular diagnostic methods, from chromosomal microarray to whole exome sequencing, routine clinical genetic testing is now recommended for the etiological evaluation of all children with new diagnoses of global developmental delay, intellectual disability (ID), or autism spectrum disorder (ASD) [1–4]. This surge in genetic testing has facilitated the identification of pathogenic rare genetic variants and, with the ascertainment of subgroups of individuals with shared variants, the identification of clinically meaningful genetic syndromes [5]. To date, detailed developmental and behavioral characterization of these syndromes has lagged behind the genetic diagnoses, leaving considerable uncertainty regarding developmental trajectories, prognosis, and recommended treatment options for these disorders, despite having a molecular diagnosis. Interventions remain broad in focus, targeting the neurodevelopmental diagnoses (such as ASD or ID) rather than specific features of cognition or social communication that may define a particular molecularly defined syndrome. This considerable dissociation between the precision of genetic testing and the imprecision of clinical treatment may be addressable and represents a critical challenge in neurodevelopmental disorders. Improved and precise behavioral characterization of genetic syndromes associated with ID and ASD can inform not only prognosis but also treatment, with the ultimate goal of facilitating the discovery of targeted, mechanism-based interventions that may improve individual outcomes.

One of the most common chromosomal abnormalities associated with ASD and ID is the duplication of chromosome 15q11.2-q13.1 (Dup15q syndrome). This region includes the imprinted Prader-Willi/Angelman syndrome critical region (PWACR) as well as several genes critical for brain development and synaptic function, such as ubiquitin protein ligase E3A (*UBE3A*), small nuclear ribonucleoprotein polypeptide N (*SNRPN*), and three GABA_A_ receptor genes (*GABRB3*, *GABRA5*, and *GABRG3*). Dup15q syndrome includes two primary types of duplications of 15q11.2-13.1: (1) an isodicentric chromosome 15 (idic(15)) that results in two additional maternally derived copies on a supernumerary chromosome that includes 15p and the proximal region of 15q11, most commonly leading to four copies of the region, or (2) an interstitial 15q duplication in which one extra copy of the 15q11.2-q13.1 region occurs on the same chromosome arm, typically resulting in three copies of the region, and has an overall milder phenotype [6–9].

### Neurodevelopmental characteristics of Dup15q syndrome

Systematic genetic screening in large cohorts of patients with ASD has revealed a high prevalence of Dup15q syndrome, with rates of 0.25–3 % depending on sample ascertainment [10–14]. The neurodevelopmental “syndrome” of idic(15) has been characterized by a constellation of clinical features including mild to profound ID, central hypotonia resulting in motor delays, mild to profound language impairment, and impairments in social communication [6, 15–26]. Although exhibiting a typically milder clinical phenotype, interstitial Dup15q also has been associated with ASD, hypotonia, and moderate ID to no general cognitive impairment [27]. Epilepsy often develops early in infancy, with rates of 63 % in idic(15) and 16 % in interstitial duplications [28, 29]. Considerable heterogeneity exists in neurodevelopmental outcomes, which may reflect whether they are interstitial or supernumerary, rare cases of more than expected duplication (e.g., interstitial duplication vs. rare interstitial triplication), additional rare or common genetic variation, timing and severity of epilepsy, or other unidentified factors [18, 26]. Although children with idic(15) demonstrate more cognitive and behavioral impairment than those with interstitial duplications, no studies have directly compared these groups with regard to development and behavior.

Most of the clinical insights gained into Dup15q syndrome have resulted from retrospective chart reviews (e.g., [25]) or case series of individual patients (e.g., [30]) and, therefore, have mostly focused on categorical diagnoses and general descriptions of developmental domains. In the largest retrospective study of patients with Dup15q syndrome, Al Ageeli and colleagues [25] reviewed 30 cases (50 % of each duplication type) and found that 77 % met the criteria for developmental delay, while 74 % had a diagnosis of ASD.

The only prospective study of children with Dup15q syndrome focused on a cohort of children with interstitial 15q11.2-q13.1 duplications. The investigators performed standardized diagnostic testing for ASD (using the Autism Diagnostic Observation Schedule (ADOS)) in order to identify genotype-phenotype relationships, and they found that 9/9 maternally derived duplications met criteria for ASD or autism and 2/4 paternally derived duplications met criteria for ASD or autism [27].

Several questions remain unanswered in the developmental and behavioral characterization of children with 15q11.2-q13.1 duplications. First, while a large proportion of children with Dup15q syndrome meet the diagnostic criteria for ASD, are there distinctive behavioral and developmental features in this cohort that are not captured by a categorical diagnosis, particularly in the domain of social communication and adaptive function? Second, is there variability within the social communication and adaptive function of this cohort and, if so, does it relate to the duplication type or epilepsy status? To begin to address these questions, we examined the social communication, adaptive and cognitive skills in a clinic-referred sample of children with Dup15q syndrome of both duplication types. In order to identify distinctive developmental and behavioral features, we compared characteristics of children with Dup15q syndrome to a chronological age- and mental age-matched cohort of children with non-syndromic ASD. We then examined the variability in behavior and development within the Dup15q group by comparing children by duplication type and by presence of epilepsy.

Based on data from the case reports described earlier, we hypothesized that we would identify a distinct developmental profile in children with Dup15q syndrome defined by expressive language and motor impairment with relatively stronger social communication skills compared to a non-syndromic ASD comparison group, but that there would be considerable heterogeneity in function based largely on duplication type and presence of epilepsy.

## Methods

### Participants

Participants included 13 children with Dup15q syndrome ages 22 months–12 years and 13 children with non-syndromic ASD (defined as children without a molecular genetic diagnosis) matched on chronological age and closest available overall developmental quotient (DQ). Children with Dup15q syndrome were recruited from the national Dup15q Alliance and the UCLA Dup15q clinic. Given the rarity of the disorder, there were no exclusionary criteria for the Dup15q participants. All children with a confirmed genetic diagnosis, based on clinical genetics reports, were included in the study. There were no data available on parent of origin. Data for the ASD comparison group was selected from existing data from research studies in the UCLA Center for Autism Research and Treatment. Exclusionary criteria for the ASD group included the diagnosis of epilepsy or a known genetic disorder.

### Ethics, consent, and permissions

All research was approved by the UCLA Institutional Review Board (IRB#14-001180) and all participants provided consent for their data to be used for related research.

### Procedures and measures

Children with Dup15q syndrome were assessed over a 2-day period. Parents reported on their child’s development through interviews and survey forms. The assessment battery included a variety of measures to assess cognition, language, adaptive behavior, motor skills, behavior problems, and social communication characteristics. Due to the range in age and abilities of the participants, multiple measures were used in each domain. Standard scores and DQ scores (calculated based on age equivalent scores) were used to facilitate comparison across assessments (Table 1).

**Table 1:** Assessment domains and measures

Domain	Measure	Variables
Cognition and language	Mullen Scales of Early Learning Leiter-R Stanford-Binet-Fifth Edition Preschool Language Scales-Fifth Edition	Standard scores IQ/DQ
Adaptive behavior	Vineland Adaptive Behavior Scales	Standard scores DQ
Motor skills	Mullen Scales of Early Learning Vineland Adaptive Behavior Scales	Standard scores DQ
Autism characteristics	Autism Diagnostic Observation Schedule-Second Version: Modules T, 1, 2, 3	Raw scores CSS

*The Mullen Scales of Early Learning* (MSEL) [31]. The MSEL is an assessment of general cognition and development designed for infants and young children with developmental ability below the age of 5 years. The MSEL yields standard and age equivalent (AE) scores for receptive and expressive language, visual reception, and gross and fine motor skills and can be used to calculate DQ scores.

*Leiter International Performance Scales-Revised* (Leiter-R) [32]. The Leiter-R is a nonverbal assessment of general cognitive ability often used to assess individuals with cognitively delay and/or limited expressive language ability. The assessment yields a developmental age equivalent and a non-verbal IQ score.

*Stanford-Binet Intelligence Scales-Fifth Edition* (SB-5) [33]. The Stanford-Binet is an assessment of intelligence and cognitive abilities, and it provides a full-scale IQ, verbal, and non-verbal IQ scores (VIQ; NVIQ).

*Preschool Language Scales-Fifth Edition* (PLS-5) [34]. The PLS-5 is a developmental language assessment. The PLS-5 yields standard scores and age equivalent scores for auditory comprehension and expressive communication, and it can be used to calculate verbal DQ.

*The Autism Diagnostic Observation Schedule-Second Version* (ADOS-2) [35, 36]. The ADOS-2, a semi-structured behavioral observation, is the gold standard instrument for confirming a clinical diagnosis of ASD. Four standard modules (1–4) plus an additional Toddler module (ADOS-T) are available and are chosen based on the child’s age and language level. ADOS items are scored on a 4- or 5-point scale, using the following scoring conventions: 0 = no abnormalities observed; 1 = subtle or occasional abnormalities; 2 = clearly abnormal, consistent with ASD characteristics; 3 = clearly abnormal to a marked degree; and 4 = skill is completely absent (no words or vocalizations; only applies to the item “overall level of language”) [35]. Some items include a “missing” code (e.g., 8), which can be used when the child lacks the opportunity to display the skill. Items coded as missing are converted to a score of 0 [35]. The ADOS yields numerical scores in the domains of social communication and repetitive behaviors. For modules 1–4, a calibrated severity score (CSS) can also be calculated, which can be used to compare scores across modules 1–4 [37]. The ADOS-T is designed to assess for a clinical presentation of ASD or other pervasive developmental disorders in young infants and toddlers under age 30 months. The scores result in categorization of risk for ASD but do not yield a CSS. In this sample, modules 1, 2, and Toddler were used.

*Vineland Adaptive Behavior Scale-II* (VABS-II) [38]. The VABS-II is a semi-structured interview conducted with the parent and assesses four domains of adaptive behavior: (1) communication, (2) daily living skills, (3) socialization, and (4) motor skills. The VABS-II yields standard scores and age equivalent scores. Because many participants in the Dup15q group performed at or near the floor on the motor domain, AE scores were used to compute DQ scores for fine and gross motor subscales. AE scores have been recommended as more accurate alternatives to VABS standard scores when assessing significantly delayed children [38, 39].

### Data analytic plan

All raw scores were converted to standard scores or developmental quotient scores to facilitate comparisons. Group level descriptive comparisons (ASD vs. Dup15q; idic vs. interstitial; epilepsy vs. no epilepsy) were performed using independent samples *t* tests. Group comparisons were performed for the following variables: chronological age, verbal developmental quotient (VDQ), non-verbal developmental quotient (NVDQ), ADOS CSS, and VABS-II domains. Item level analysis of the ADOS was performed using a repeated measures analysis of variance, with group as the between-subjects factor and ADOS item as the within-subjects factor. Analysis focused on differences in the mean scores as well as the pattern of score distribution across items. Because items vary across ADOS modules, this analysis was only performed with participants who received module 1 (*N* = 9). Planned post hoc comparisons were then carried out on each item in the reciprocal social interaction (RSI) subscale, to further investigate group differences in social function. Because of the small sample size and descriptive nature of this item level analysis, correction for multiple comparisons was not performed. For all analyses with missing data in either the Dup15q or ASD group, the corresponding individuals were removed from the other group, to preserve matching. More details for each analysis are provided in the “Results” section.

## Results

Table 2 presents the descriptive clinical data regarding each participant in the Dup15q group, including the identity of the direct assessments that were completed for each assessment domain. Ten children (77 %) had idic(15) while three children (23 %) had an interstitial duplication. Four children (31 %; all idic) had active epilepsy requiring up to three medications. The children with epilepsy ranged in age from 36 to 144 months; three (75 %) were female. Two (50 %) of the children had a history of infantile spasms and current complex partial seizures, while the other two had onset of generalized tonic clonic seizures in late childhood. No participants in the ASD group had a known genetic condition or active epilepsy.

**Table 2:** Dup15q syndrome participants

Participant	Age (months)	Gender	Duplication^a^	Epilepsy (active)	Antiepileptic medication^b^	ADOS calibrated severity score	ADOS module	Developmental quotient	Cognitive assessment
1	22	F	Idic	No	N/A	N/A	T	42.05	MSEL
2	36	F	Idic	Yes	Lev, Zns, Cbz	N/A	AOSI	4.86	MSEL
3	37	M	Idic	No	N/A	5	1	67.57	MSEL
4	38	M	Idic	No	N/A	8	1	21.71	MSEL
5	54	F	Idic	No	N/A	8	1	40.74	MSEL
6	56	M	Idic	No	N/A	7	1	46.88	MSEL
7	94	F	Idic	No	N/A	9	2	45 (IQ)	SB-5
8	122	M	Idic	Yes	Cbz, Ruf	6	1	8.87	MSEL
9	144	F	Idic	Yes	Ltg	6	1	52 (NVIQ)	Leiter-R
10	144	F	Idic	Yes	Lev	6	1	9.2	MSEL
11	48	F	Int	No	N/A	5	2	102.6	MSEL
12	50	M	Int	No	N/A	6	1	23.5	MSEL
13	54	M	Int	No	N/A	10	1	16.2	MSEL

*Idic* isodicentric, *Int* interstitial, *Lev* leviteracitam, *Cbz* clobazam, *Zns* zonisamide, *Ruf* rufinamide, *Ltg* lamotrigine, *N/A* not applicable

^a^Duplication

^b^Medications

### ASD vs. Dup15q: cognition and language

Cognition and language skills were assessed using a variety of standardized measures. Several participants required the MSEL and PLS-5 despite being older than the normal chronological age range due to their developmental delays. As a result, age equivalent scores were used to create DQ scores for these domains (consistent with published recommendations, e.g., [40]). For participants who received the Leiter-R and SB-5, IQ scores were generated. Prior research has demonstrated high convergent validity across these measures [41].

**Fig. 1:**
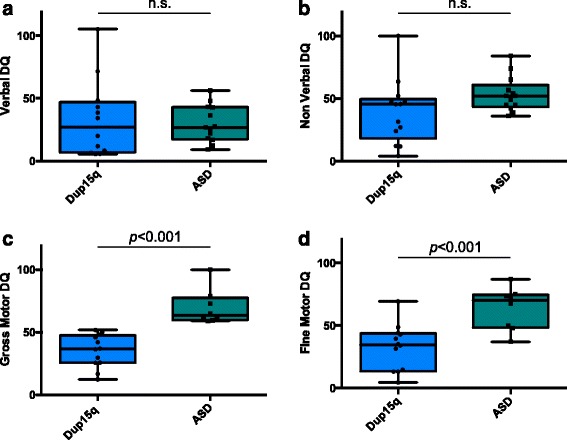
Box plots of developmental quotient scores by group. Score distributions for ASD and Dup15q groups, showing significant differences in gross and fine motor scores. **a** Verbal DQ (*M*(SD)*:* ASD *=* 29.69(14.59); Dup15q = 33.24(30.89); *p =* 0.7). **b** Non-verbal DQ (verbal DQ *M*(SD): ASD *=* 53.38(13.92); Dup15q = 39.34(25.50); *p =* 0.09). **c** Gross motor DQ (verbal DQ (*M*(SD): ASD *=* 69.81(14.06); Dup15q = 34.43(13.61); *p <* 0.001) and **d** fine motor DQ (verbal DQ (*M*(SD): ASD *=* 63.87(16.97); Dup15q = 29.22(15.11); *p <* 0.001)

Confirming matching procedures, based on independent samples *t* tests, the Dup15q group did not significantly differ from the ASD group in terms of chronological age, verbal, or non-verbal IQ/DQ scores, as shown in Table 3 (Fig. 1).

**Table 3:** Assessment domain scores for Dup15q and ASD groups

	Dup15q (idic *N* = 10, interstitial *N* = 3, epilepsy *N* = 4)			ASD (epilepsy *N* = 0)			
	*N*	*M* (SD)	Range	*N*	*M* (SD)	Range	*T*, *p*
Chronological age (months)	13	69.15 (42.22)	22–144	13	64.77 (34.58)	22–125	−0.29, 0.78
Verbal DQ	12	33.24 (30.89)	5.56–105.21	13	29.69 (14.59)	9.18–56.25	−0.37, 0.71
Non-verbal DQ	13	39.34 (25.50)	4.17–100	13	53.38 (13.92)	36–84	1.74, 0.09
Total DQ	13	37.01 (27.55)	4.86–102.6	13	41.53 (12.83)	26.4–65.1	0.54, 0.60
ADOS CSS	11	6.91 (1.64)	5–10	11	8.5 (1.57)	5–10	2.26, 0.03*
VABS-II							
Gross motor DQ	12	34.43 (13.61)	12.3–51.79	9	69.81 (14.06)	58.93–100	5.9, <0.001**
Fine motor DQ	12	29.22 (15.11)	4.55–48.64	9	63.87 (16.98)	37.04–87.03	5.2, <0.001**
Communication	12	54.67 (18.20)	36–97	12	62.50 (7.79)	52–76	1.37, 0.18
Daily living skills	12	55.17 (14.08)	38–77	12	66.08 (10.26)	55–91	2.17, 0.04*
Socialization	12	58.92 (14.79)	36–79	12	60.33 (7.79)	48–75	0.29, 0.77

*p=<0.05

**p=<0.01

### ASD vs. Dup15q: motor skills

The VABS-II parent survey form was returned for 12 participants in the Dup15q group. VABS-II motor domain scores were available for nine participants in the ASD group, due to the fact that this section was not collected for children with ASD over age 7. To preserve matching, the corresponding participants were removed from the Dup15q group for these analyses, leaving a comparison of nine subjects per group. Parent report of motor skills differed significantly between groups, for both gross motor DQ (Dup15q *M* = 35.38, ASD *M* = 70.21; *t* = 5.9, *p* < 0.001) and fine motor DQ (Dup15q *M* = 30.03, ASD *M* = 66.5; *t* = 5.2, *p* < 0.001). In addition to parent report, motor skills were directly assessed (using the MSEL) in 11 children with Dup15q syndrome. Direct assessment and parent report of motor skills did not significantly differ for either fine (*t* = 1.08, *p* = 0.31) or gross motor (*t* = 0.92, *p* = 0.38), indicating that parent report of motor skills accurately reflected the child’s abilities as observed by a trained assessor.

### ASD vs. Dup15q: adaptive skills

**Fig. 2:**
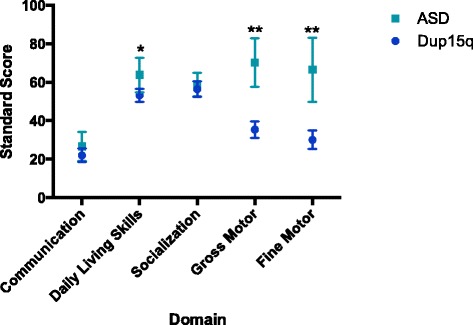
VABS-II domain scores by group. Average VABS-II subscale scores by group, showing significant differences in the DLS domain (Dup15q *M* = 53.18, ASD *M* = 63.82; *t* = 2.41, *p* = 0.03), gross motor DQ (Dup15q *M* = 35.38, ASD *M* = 70.21; *t* = 5.9, *p* < 0.001), and fine motor DQ (Dup15q *M* = 30.03, ASD *M* = 66.5; *t* = 5.2, *p* < 0.001), * significant at *p*<.05, ** significant at *p<*.01

The VABS-II was administered to assess parent report of adaptive behavior across four domains: communication, daily living skills (DLS), socialization, and motor skills (reported above), and was returned for 12 participants in the Dup15q group. Consistent with the results from direct assessment, parent report of communication skills did not significantly differ between groups for either receptive (Dup15q *M* = 17.87, ASD *M* = 26.63; *t* = 1.28, *p* = 0.21) or expressive language (Dup15q *M* = 25.99, ASD *M* = 26.64; *t* = 0.10, *p* = 0.93). The communication domain also includes a “written” subscale, which was not analyzed due to the fact that the majority of participants in both groups did not yet have any written skills. There were also no significant group differences in the socialization domain (Dup15q *M* = 57.09, ASD *M* = 59.45; *t* = 0.49, *p* = 0.63). Children with Dup15q syndrome did have significantly lower scores in the DLS domain as compared to ASD (Dup15q *M* = 53.18, ASD *M* = 63.82; *t* = 2.41, *p* = 0.03) (Fig. 2).

### ASD vs. Dup15q: autism symptomatology

Children in both groups were assessed using the ADOS, with the appropriate module based on their chronological age and language level. In the ASD group, all participants were assessed with module 1. In the Dup15q group, nine participants were assessed with module 1, two with module 2, and one with the ADOS-T. One child with Dup15q syndrome could not be assessed with the ADOS due to the degree of developmental delay and non-ambulatory status, and the ADOS score for their ASD match was not included. In both groups, one participant met the ADOS cutoff for ASD while the remaining participants met the cutoff for Autism.

CSSs, which allow for comparison across modules, were used to evaluate group differences. Because the ADOS-T does not yield a CSS, one child in the Dup15q group and the corresponding ASD match were removed from this analysis, resulting in 11 participants per group. Although the range of CSS was the same in both groups (5–10), the Dup15q participants had a significantly lower average CSS (lower autism severity) than the idiopathic ASD group (Dup15q *M* = 6.91, ASD *M* = 8.45; *t* = 2.26, *p* = 0.03). This mean difference of 1.5 points is clinically meaningful, as the CSS only has a range of 10 points [37].

To further investigate this difference in autism characteristics between groups, individual ADOS item scores were compared through a repeated measures analysis of variance. Because items vary across modules, only individuals who received an ADOS module 1 were included in this analysis (*N* = 9). The group difference in CSS was confirmed for the module 1 participants (Dup15q *M* = 6.89, ASD *M* = 8.67; *t* = 2.81, *p* = 0.01).

**Fig. 3:**
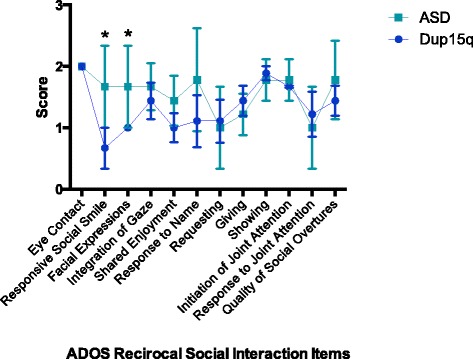
ADOS reciprocal social interaction item scores by group. Average ADOS RSI item scores by group, showing significant differences for two items: “responsive social smile” (ASD *M =* 1.67, Dup15q *M =* 0.67; *t =* 2.27, *p =* 0.04) and “directs facial expressions to others” (ASD *M =* 1.67, Dup15q *M =* 1; *t* = 2.31, *p* = 0.04), * significant at *p*<.05

The repeated measures ANOVA revealed no main effect of group (*F* = 0.91, *p* = 0.35) or group by item interaction (*F* = 0.72, *p* = 0.84), indicating that the pattern of scores across items was consistent between groups. Because our ability to detect statistically significant differences between groups was limited by the small sample size, planned post hoc analyses were carried out to test for group differences in individual items in the RSI subscale. Based on our hypothesis of a relative strength in social skills in the Dup15q group, as well as visual inspection of the data, we expected that the RSI items would be the most likely to reflect group differences. As can be seen in Fig. 3, children with Dup15q syndrome demonstrated lower (less impaired) scores on many of the RSI items. Given the small sample size and descriptive nature of this analysis, we did not correct for multiple comparisons. *t* tests revealed group differences for the items “responsive social smile” (*t =* 2.27, *p =* 0.04) and “facial expressions directed to others” (*t* = 2.31, *p* = 0.04), with higher abilities in those with Dup15q syndrome. Of note, while all participants received a score of 2 on “eye contact” (item 9), indicating that they did not consistently use well-integrated eye contact to communicate social intention, the children in the Dup15q group nevertheless showed relative strengths in responding to social smiles and direction of facial expressions to others, both of which are skills that involve directing gaze towards another person.

### ASD vs. Dup15q: skill relationships

In order to characterize the pattern of skill relationships in each group, correlational analyses were performed to determine how DQ, ADOS CSS, adaptive behavior, and motor skills were related to each other (Table 4). In cases of missing data, participants were excluded pair-wise from analyses.

**Table 4:** Relationships across skill domains by group

	ASD						Dup15q					
Skill domain	1	2	3	4	5	6	1	2	3	4	5	6
1. VDQ	–						–					
2. NVDQ	0.62*	–					0.97**	–				
3. ADOS CSS	0.72**	0.35	–				−0.46	−0.34	–			
VABS-II												
4. Communication	0.65*	0.44	0.26	–			0.96**	0.91**	0.25	–		
5. DLS	0.12	0.27	0.27	0.24	–		0.90**	0.82**	0.27	0.86**	–	
6. Socialization	0.32	0.28	0.41	0.58*	0.71*	–	0.78**	0.63*	0.08	0.72**	0.89**	–
7. Motor	−0.29	0.17	0.33	0.25	0.89**	0.60	0.85**	0.78**	0.56	0.77**	0.80**	0.66*

*p < 0.05; **p < 0.01

Overall, results indicate that skills are closely associated across domains within the Dup15q group, while there are far fewer significant associations in the ASD group. Within the Dup15q group, verbal DQ was related to all four VABS-II domains, while NVDQ was related to the communication, DLS, and motor skills domains. Motor skills were associated with VDQ and NVDQ, as well as VABS-II DLS. In contrast, there were few significant associations in the ASD group. For children with ASD, VDQ was only associated with VABS-II communication and ADOS CSS. NVDQ and motor skills showed no significant associations. ADOS CSS was not significantly related to adaptive behavior domains in either group. Overall, children in the Dup15q group showed a profile marked by strong correlations across ability domains. This pattern was not evident in the ASD group, despite the fact that ASD participants were matched on overall developmental level.

### Comparisons by duplication type and epilepsy

**Fig. 4:**
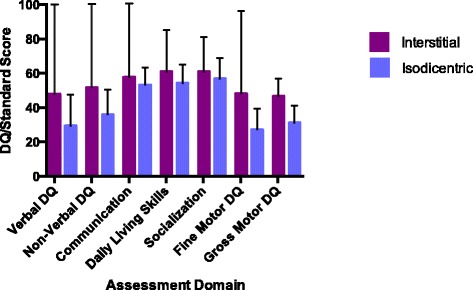
Assessment domain scores by duplication type. Assessment domain scores by duplication type, showing no significant differences

In order to examine heterogeneity within the Dup15q group, participants were compared based on duplication type (idic(15) vs. interstitial) and diagnosis of epilepsy. Verbal and non-verbal DQ, fine and gross motor skills, and ADOS severity scores did not differ between children with idic(15) (*N* = 10) and interstitial (*N* = 3) duplication types. It was notable that the interstitial sample in this study was, on average, more impaired than those children described by Urraca and colleagues [27] and, therefore, may reflect a less representative sample of subjects with interstitial Dup15q. While the mean scores across measures were higher in the interstitial group (consistent with prior research), the small sample size and unbalanced groups precluded the detection of statistically significant group differences (Fig. 4).

**Fig. 5:**
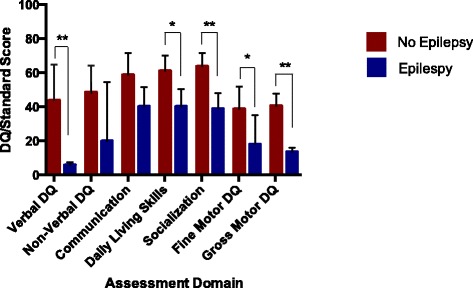
Assessment domain scores by epilepsy status. Assessment domain scores by epilepsy status, showing significant differences in VDQ (epilepsy *M =* 6.12, no epilepsy *M =* 43.91; *t* = 3.56, *p* = 0.007), VABS-II DLS (epilepsy *M =* 40.33, no epilepsy *M =* 61.20; *t =* 2.68, *p =* 0.03), VABS-II social (epilepsy *M =* 39.00, no epilepsy *M =* 63.9; *t =* 4.21, *p* < 0.001), fine motor (epilepsy *M =* 13.71, no epilepsy *M =* 38.84; *t* = 2.47, *p* = 0.03), and gross motor (epilepsy *M =* 18.22, no epilepsy *M =* 40.67; *t* = 3.83, *p* = 0.003), * significant at *p*<0.05, ** significant at *p*<0.01

Significant differences in cognition and adaptive skills did emerge based on the presence or absence of epilepsy. All children in the epilepsy group had active seizures, with 50 % having a history of infantile spasms and subsequent complex partial seizures, while 50 % were diagnosed with generalized epilepsy around puberty. Therefore, while the sample size was small, the epilepsy characteristics represent the range of epilepsy types found in idic (15). Compared to children without epilepsy (*N* = 9), those with epilepsy (*N* = 4) showed significantly lower verbal DQ (epilepsy *M =* 6.12, no epilepsy *M =* 43.91; *t* = 3.56, *p* = 0.007), fine motor skills (epilepsy *M =* 13.71, no epilepsy *M =* 38.84; *t* = 2.47, *p* = 0.03), and gross motor skills (epilepsy *M =* 18.22, no epilepsy *M =* 40.67; *t* = 3.83, *p* = 0.003). Children without epilepsy also had significantly higher scores on two VABS-II domains: DLS (epilepsy *M =* 40.33, no epilepsy *M =* 61.20; *t =* 2.68, *p =* 0.03) and socialization (epilepsy *M =* 39.00, no epilepsy *M =* 63.9; *t =* 4.21, *p* < 0.001) (Fig. 5).

All four participants with epilepsy had idic(15) duplication types. Because of the potential confound of duplication type, the epilepsy group comparisons were repeated with only the idic(15) group (epilepsy *N* = 4, no epilepsy *N* = 6). The results remained consistent with the full group comparison reported above, with additional group differences in NVDQ and VABS-II communication. Within the idic(15) group, participants with epilepsy had significantly lower VDQ (*t* = 4.44, *p* = 0.007), NVDQ (*t* = 2.67, *p* = 0.028), VABS-II communication (*t* = 2.63, *p* = 0.02), DLS (*t* = 2.67, *p* = 0.02), socialization (*t* = 4.83, *p* = 0.003), fine motor (*t* = 4.62, *p* = 0.004), and gross motor abilities (*t* = 6.03, *p* = 0.001). However, the ADOS CSS did not significantly differ between groups (*t* = 1.55, *p* = 0.17).

## Discussion

In this study, we sought to examine the cognitive, adaptive behavioral, and social communication profiles of children with Dup15q syndrome in order to identify features that can distinguish this syndrome from non-syndromic ASD and to understand the phenotypic variability within the Dup15q syndrome. Inclusion of a non-syndromic ASD comparison group was crucial for interpreting the skill profile observed in children with Dup15q syndrome, especially with regard to social-communication skills, given the high rate of ASD comorbidity. Several key themes have emerged that necessitate larger scale efforts in targeted and prospective examination of development in this high-risk population. First, all children with Dup15q syndrome demonstrated ADOS scores in the ASD/autism range but exhibited a distinctive developmental profile compared to a matched sample of children with non-syndromic ASD. This profile was characterized by significant delays in motor skills and adaptive function with relative strengths in isolated social communication skills that may relate to social interest. Secondly, children with Dup15q syndrome demonstrated a distinctive and strong association between motor skills and both language and social function, leading to the question of whether early motor delays contribute to the development of social communication impairments. Finally, children with epilepsy demonstrated significantly greater impairment across cognitive and developmental domains compared to children without epilepsy. The epilepsy characteristics of this small group ranged from severe infantile spasms to occasional generalized tonic clonic seizures that emerged in late childhood in concordance with previous reports [29].

### Adaptive, motor, and social communication skills

Previous research has suggested that individuals with ASD show a distinct skill profile on the VABS-II, with greatest impairment in the socialization skills with relatively spared motor and daily living skills [42, 43]. In our study, children with non-syndromic ASD demonstrated this well-described pattern of relative strengths and weaknesses on the VABS-II. By contrast, children with Dup15q syndrome did not demonstrate evidence of spared motor and daily living skills on the VABS-II. Their adaptive skills remained relatively impaired across domains, with ratings on daily living and motor skills significantly lower than the children in the ASD group. This pattern of “flat” scores across domains is consistent with previous studies of adaptive skills in children with ID [44–46] and indeed, impairments in adaptive behavior (as opposed to cognition alone) remain central to the diagnostic criteria for ID [47]. Moreover, motor skills were significantly correlated with language, non-verbal cognition, and daily living skills in the Dup15q group, and these relationships were not present in the ASD group. It is interesting to note that these significant differences in motor skills and adaptive function emerged despite participants being matched on the overall developmental quotient. This highlights the distinction between general cognition and adaptive behavior (practical skills employed in everyday life) and suggests that children with Dup15q syndrome may have specific difficulty learning the skills necessary for daily activities.

All of the children with Dup15q syndrome met the ADOS score cutoffs for ASD or autism. However, they demonstrated lower overall ASD severity than the non-syndromic ASD group. Further investigation revealed that children with Dup15q syndrome showed relative strength in the social domain, with significantly better performance on two items: "responsive social smile" and "facial expressions directed to others". These items reflect specific instances of social communication during the administration of the ADOS. To receive a “passing score” (score of 0) on response to social smile, the child must immediately smile back to the adult who is directing a smile towards them. This item is generally administered with the adult directly in front of the child. The item "facial expressions directed to others" is somewhat broader but still involves displaying isolated instances of a skill. To receive a passing score on this item, a child must spontaneously shift gaze to direct a range of facial expressions to the adult for the purpose of communication (e.g., looking to the adult with a surprised expression to express surprise when the jack-in-the-box pops up). Unlike "social smile", this behavior must be initiated by the child but is still comprised of discrete instances. Interestingly, their performance on the "eye contact" item was poor and did not differ from the ASD group. Unlike “social smile” and “facial expressions,” the "eye contact" item is a summary score based on the child’s performance across the entire assessment and is forced to be coded as normal or abnormal, not allowing an intermediate code on this specific item. This may suggest that children with Dup15q syndrome demonstrate clear and specific instances of social interest during the ADOS but that overall, their eye contact remains infrequent and poorly modulated. Taken together, these findings indicate that while children with Dup15q syndrome are likely to meet criteria for an ASD diagnosis, they exhibit relative strengths in social interest and responsiveness through behaviors that occur discretely. As discussed below, one may ask whether an underlying social motivation is present that may be impeded or disrupted by the profound delays in other developmental domains, such as motor skills (such as difficultly in sustained head control and low truncal tone, due to their underlying neuronal pathology). Although our sample size led us to perform statistical comparisons only of items in the reciprocal social interaction subscale, visual inspection of the data suggested that the groups showed similar patterns of performance on the communication and restricted and repetitive behaviors subscales. Future research with a larger sample size will facilitate a full comparison of all ADOS items.

### Cause or common pathways?

The strong association between motor and social communication skills in children with Dup15q syndrome, and the relative strength in measures of social interest and responsiveness, raises a question of causality vs. common pathways in the developmental phenotype of Dup15q syndrome. It is possible, particularly given the early diagnosis of hypotonia in infants with Dup15q syndrome [17], that early motor delays do limit or even obscure the development of early social communication skills, such as gesturing, eye contact, and even expressive language [48]. For instance, hypotonia and poor head control can undermine an infant’s ability to make adequate and sustained eye contact or to visually inspect his/her social environment, such as faces, which, in turn, can limit the infant’s ability to learn from these critical social cues. This hypothesis finds some support in research of infants at high risk for ASD, with risk conferred by having an older sibling with ASD, in which gross motor delays at age 6 months were associated with a later ASD diagnosis in those children who demonstrated the most severe autism symptoms [49]. An alternate possibility, however, and one articulated in a recent review by Krystal and State [50] is that duplications on 15q11.2-q13.1 have a more global impact on the developing nervous system which, in turn, impairs fundamental processes in brain development resulting in delays in the acquisition of skills across domains, from motor to social communication to language skills to risk for epilepsy. To truly disentangle these two processes, we need more refined measures of domain-specific function in early development, combined with structural and functional imaging that can link behavior to specific neural networks, and prospective studies of infants with the syndrome prior to the onset of delays. The latter study design becomes challenging given the fact that most children with Dup15q syndrome are diagnosed in the context of clinical sequelae of the syndrome, such as epilepsy or a neurodevelopmental disorder.

### Impact of duplication type and epilepsy

The subgroup comparisons were limited by sample size, and possible ascertainment bias, but certainly warrant further investigation. While the interstitial participants in this study did show higher average scores across domains than the children with idic(15) duplications, there were no significant differences between groups. These analyses were likely limited by the small sample size of interstitial duplications and, moreover, the fact that two thirds of the children with interstitial duplications in this sample demonstrated more severe cognitive impairment than the average participant in a larger cohort study of interstitial duplications [27]; thus, ascertainment bias may further limit interpretation.

Children with epilepsy had significantly lower cognitive and motor skill scores compared to those children without epilepsy, even when the analysis was confined to those with idic(15). The epilepsy characteristics of this group of four children did represent a range in epilepsy severity consistent with the epilepsy characteristics of children with Dup15q syndrome [29] including history of infantile spasms, onset of generalized epilepsy around puberty, as well as complex partial seizures, all requiring treatment with anti-epileptics. Notably, two of the four children in this cohort developed epilepsy in later childhood, after their developmental delays had emerged. The relationship between cognitive impairment and epilepsy in children with ASD has been well described in large cohort studies and meta-analyses [51, 52], leading to a similar question about causal relationships. While both epilepsy and developmental delay may reflect outcomes from common processes, such as excitation/inhibition imbalance from defects in GABA_A_ receptor function, seizures in the developing brain may also impact synaptic plasticity and cortical connectivity, which, in turn, results to developmental delays across domains [53, 54]. Further research that carefully tracks cognitive development, behavior, and epilepsy (from electrophysiological characteristics to clinical events) in this population is required to disentangle the effect of epilepsy from the underlying genetic variation in the severity of the developmental disability [55].

### Intervention and treatment goals

The relative strength observed in social interest and responsiveness in the context of impaired motor skills represents an important avenue for intervention. Intervention targeting joint attention and language within a play and engagement-based context has been established as effective for building language and social skills in children with ASD, including children with low IQ and minimal language [56, 57]. Similar intervention strategies with children with Dup15q syndrome could leverage their social interest and facilitate the development of further communication and social abilities. Additionally, intervention that focuses on fostering parent-child interactions may ultimately promote additional opportunities for children to engage in these skills throughout the day, thus increasing their opportunities for learning. Parent-mediated joint engagement-based intervention has been shown to be successful in improving social communication in toddlers with ASD [58]. Daily home routines provide an ideal context in which to promote social communication, while providing opportunities to practice motor and daily living skills. Embedding motor skills practice within an engagement-based social communication intervention has the potential to target the specific deficits observed in children with Dup15q syndrome, while building on their strength in social interest.

Given that children with epilepsy demonstrate greater cognitive impairment, behavioral intervention should be coupled with aggressive and timely treatment of seizures. Further research will be required to determine the effects of specific anti-epileptics and their timing on developmental outcomes in this cohort.

### Limitations and future directions

Although this study represents a uniquely detailed clinical characterization of a neurogenetic disorder, the sample size of 13 restricts statistical analyses and detection of significant effects that distinguish Dup15q syndrome, especially with regard to comparisons within the Dup15q group (by duplication type and epilepsy status), and precludes statistical correction for multiple comparisons. Furthermore, given that this was a clinic-referred sample, there exists an ascertainment bias towards children with greater symptom severity. Children in this cohort were diagnosed with Dup15q syndrome because of the sequelae of their genetic variation, including epilepsy, cognitive impairment, or ASD and not through a population screening of children with known ASD or a more general population sample, which would be ideal. Second, the wide range in ability and age of the participants necessitated variability of assessments across participants. Matching of mental age was better for verbal DQ than for non-verbal DQ, representing an inherent challenge in comparing groups with differing developmental profiles.

There are multiple ways that the findings in this study may be extended. An effective approach for other rare disorders has been multi-site studies to boost the sample size and statistical power needed to detect differences within subgroups of Dup15q syndrome. Prospective studies from early development will elucidate the temporal relationship between motor impairments and social communication skills, but these studies remain somewhat challenging given that the neurodevelopmental features of the syndrome usually precede the genetic diagnosis. However, with improved early developmental screening and increasingly widespread genetic testing, we will identify an increasing number of infants with Dup15q syndrome, prior to a formal ASD or ID diagnosis. Such a shift will facilitate prospective, developmentally informed studies. Finally, we must further explore through more refined measures the distinction between social behavior and social motivation in this cohort. The social motivation hypothesis in autism suggests that many children with ASD are less rewarded by social information (for review, see [59]). One could argue that the social communication deficit in Dup15q syndrome is not rooted in the lack of social motivation, rather in an inability (either due to cognitive delays or motor impairment) to sustain social interaction, or impairment in the quality rather than the amount of social interaction. Future studies should focus on social motivation by interrogating reward circuitry through measures of social attention and physiological responses to social stimuli.

## Conclusions

In conclusion, we identified a behavior profile unique to the Dup15q syndrome participants compared to ASD. This profile includes relative weakness in the areas of motor skills, facial expression, social smile, and reciprocal social interaction. These deficits were more severe in the presence of epilepsy. Research exploring the use of intervention strategies specifically targeted to the social interest strengths while addressing the significant motor deficits observed here will provide crucial information to families and professionals making decisions regarding education and therapy for children with Dup15q syndrome.

## Abbreviations

ADOS: Autism Diagnostic Observation Schedule
AE: age equivalent
ASD: autism spectrum disorder
CSS: calibrated severity score
DLS: daily living skills
DQ: developmental quotient
Dup15q: duplication of chromosome 15q11.2-q13.1
ID: intellectual disability
idic(15): isodicentric chromosome 15
IQ: intelligence quotient
Leiter-R: Leiter International Performance Scales-Revised
MSEL: Mullen Scales of Early Learning
NVDQ: non-verbal developmental quotient
PLS-5: Preschool Language Scales-Fifth Edition
PWACR: Prader-Willi/Angelman syndrome critical region
RSI: reciprocal social interaction
SB-5: Stanford-Binet Intelligence Scales-Fifth Edition
SNRPN: small nuclear ribonucleoprotein polypeptide N
UBE3A: ubiquitin protein ligase E3A
VABS-II: Vineland Adaptive Behavior Scales-II
VDQ: verbal developmental quotient
